# Supplementary material for: Development of a Novel CD26-Targeted Chimeric Antigen Receptor T-Cell Therapy for CD26-Expressing T-Cell Malignancies
Source: Cells. 2023 Aug 14;12(16):2059. doi: 10.3390/cells12162059 (PMC10453178; doi:10.3390/cells12162059)

## Slide 1
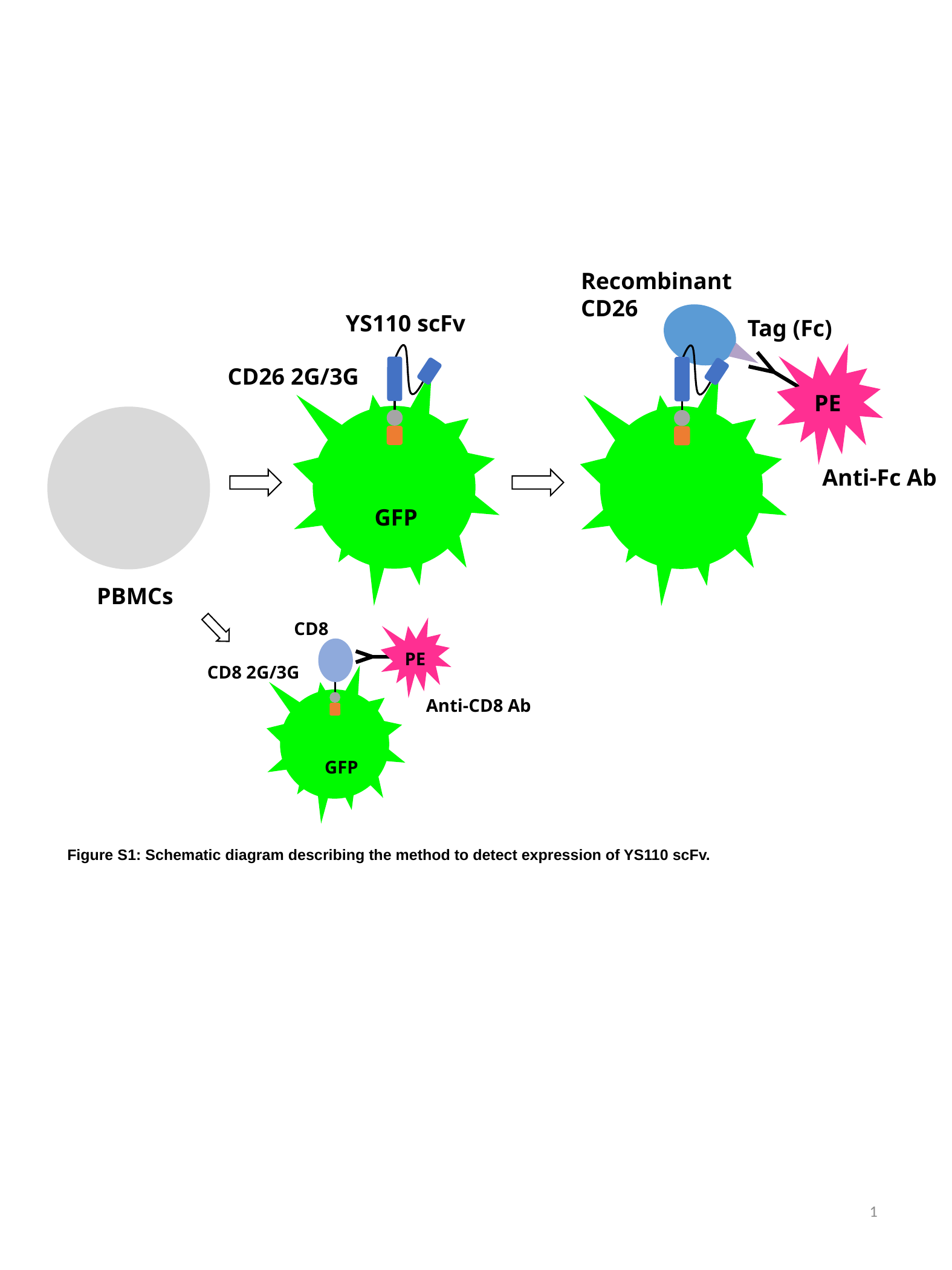

Recombinant
CD26
YS110 scFv
Tag (Fc)
CD26 2G/3G
PE
Anti-Fc Ab
GFP
PBMCs
CD8
PE
CD8 2G/3G
Anti-CD8 Ab
GFP
Figure S1: Schematic diagram describing the method to detect expression of YS110 scFv.
1

## Slide 2
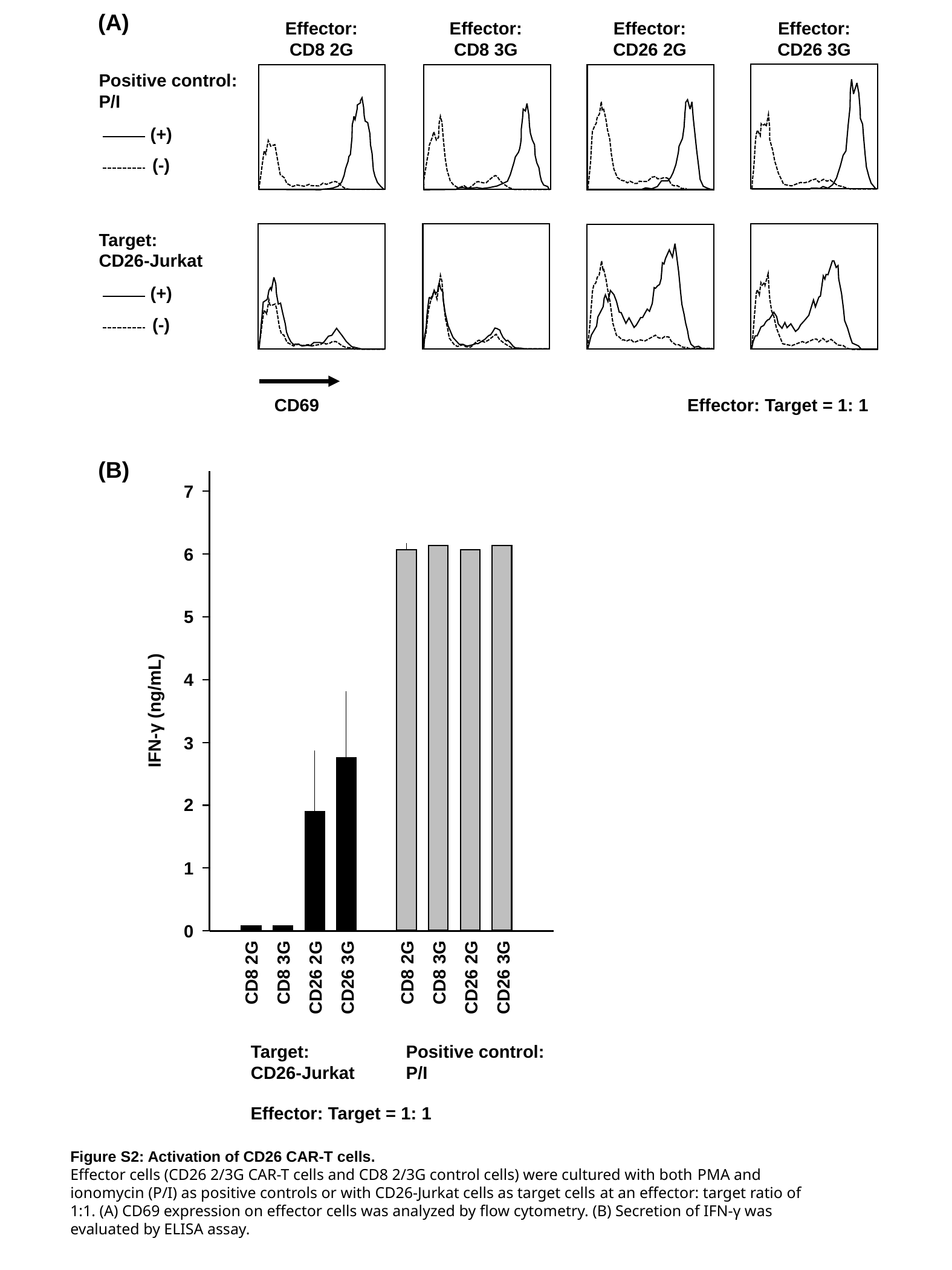

(A)
Effector:
CD8 2G
Effector:
CD8 3G
Effector:
CD26 2G
Effector:
CD26 3G
Positive control:
P/I
(+)
(-)
Target:
CD26-Jurkat
(+)
(-)
CD69
Effector: Target = 1: 1
(B)
7
6
5
4
IFN-γ (ng/mL)
3
2
1
0
CD8 2G
CD8 3G
CD8 2G
CD8 3G
CD26 2G
CD26 3G
CD26 2G
CD26 3G
Target:
CD26-Jurkat
Positive control:
P/I
Effector: Target = 1: 1
Figure S2: Activation of CD26 CAR-T cells.
Effector cells (CD26 2/3G CAR-T cells and CD8 2/3G control cells) were cultured with both PMA and ionomycin (P/I) as positive controls or with CD26-Jurkat cells as target cells at an effector: target ratio of 1:1. (A) CD69 expression on effector cells was analyzed by flow cytometry. (B) Secretion of IFN-γ was evaluated by ELISA assay.

## Slide 3
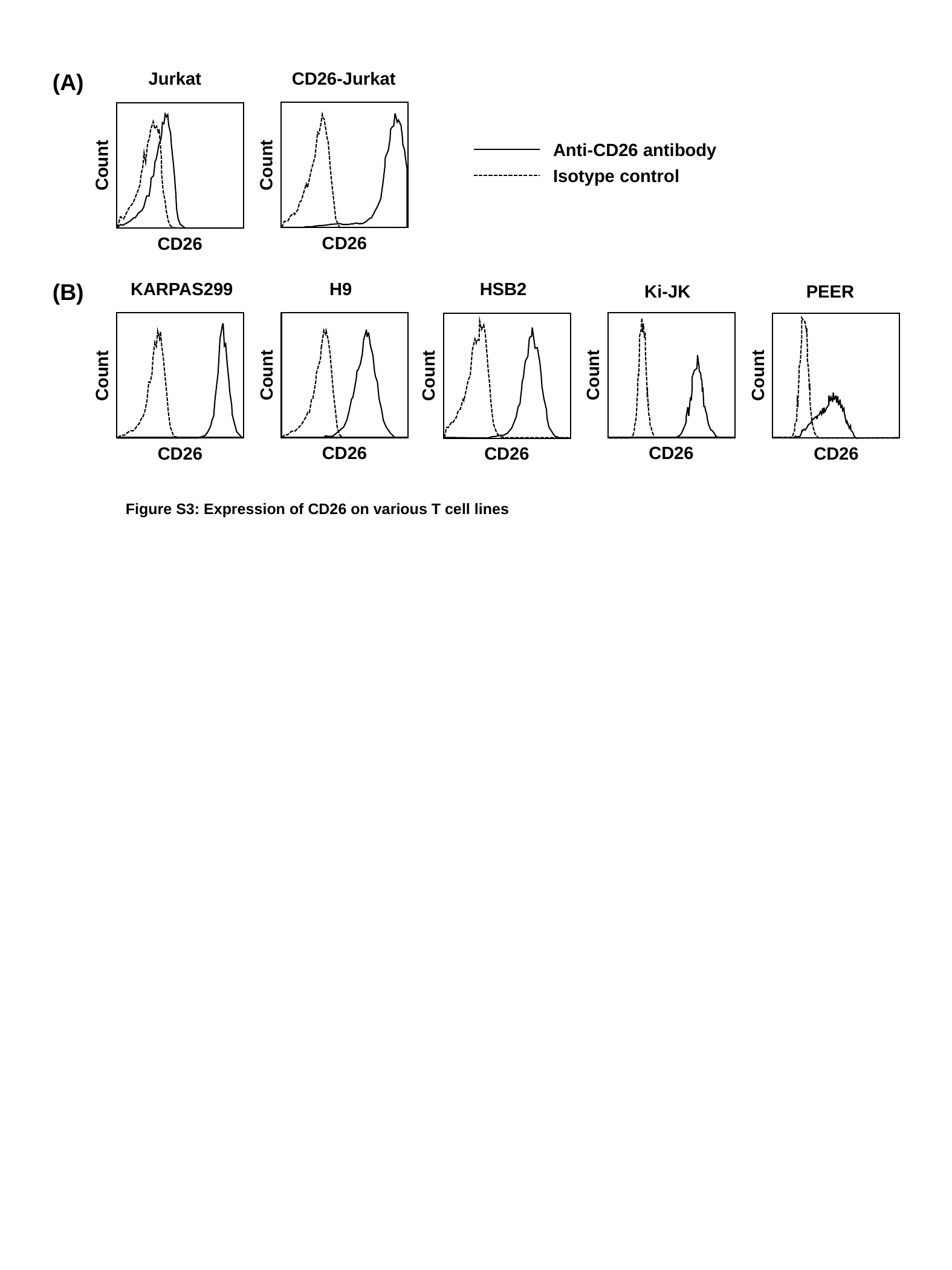

(A)
Jurkat
CD26-Jurkat
Anti-CD26 antibody
Count
Count
Isotype control
CD26
CD26
(B)
KARPAS299
H9
HSB2
Ki-JK
PEER
Count
Count
Count
Count
Count
CD26
CD26
CD26
CD26
CD26
Figure S3: Expression of CD26 on various T cell lines

## Slide 4
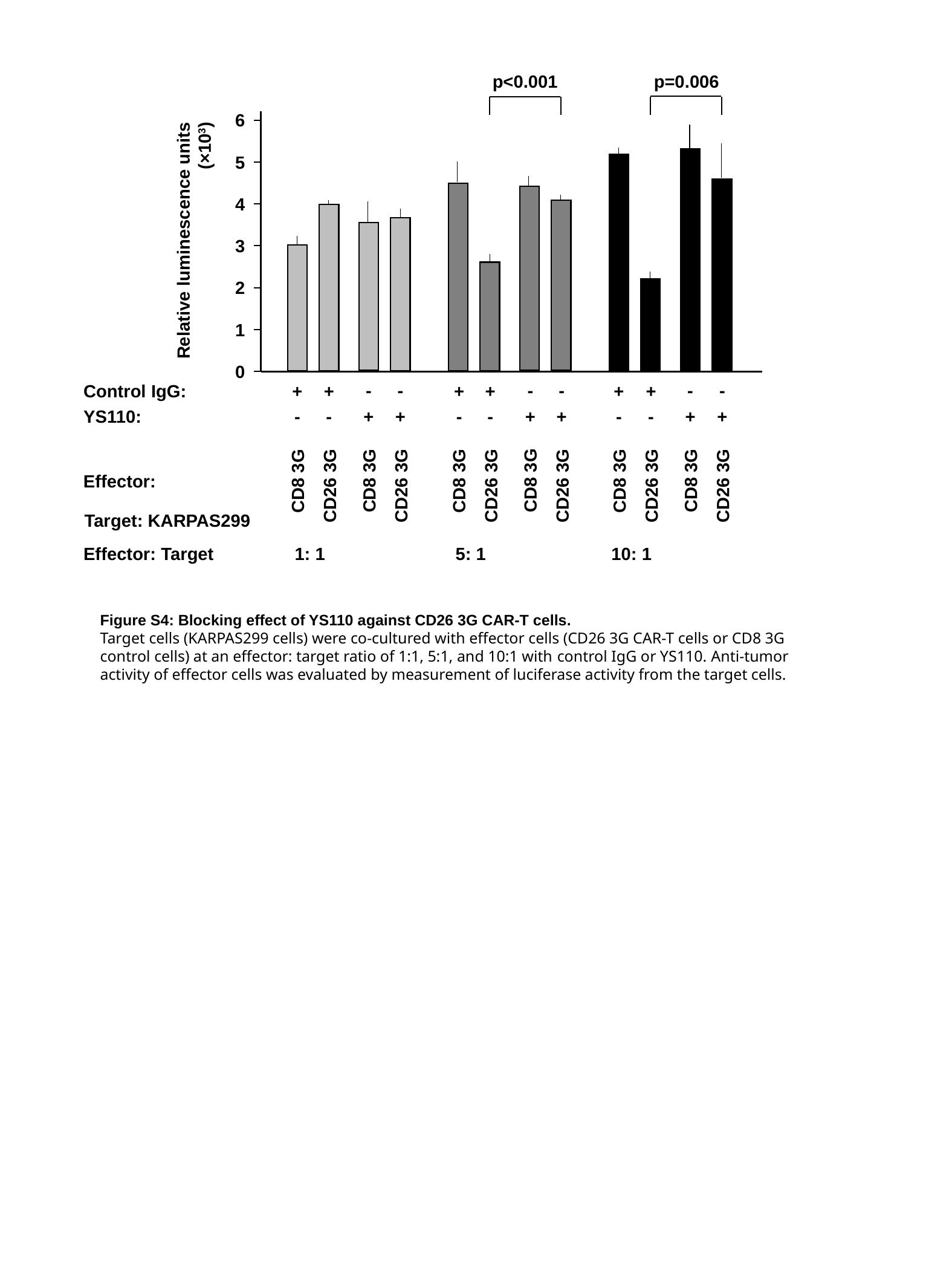

p=0.006
p<0.001
6
5
4
Relative luminescence units
(×103)
3
2
1
0
-
-
-
-
-
-
Control IgG:
+
+
+
+
+
+
+
+
+
+
+
+
YS110:
-
-
-
-
-
-
Effector:
CD8 3G
CD8 3G
CD8 3G
CD8 3G
CD8 3G
CD8 3G
CD26 3G
CD26 3G
CD26 3G
CD26 3G
CD26 3G
CD26 3G
Target: KARPAS299
Effector: Target
1: 1
5: 1
10: 1
Figure S4: Blocking effect of YS110 against CD26 3G CAR-T cells.
Target cells (KARPAS299 cells) were co-cultured with effector cells (CD26 3G CAR-T cells or CD8 3G control cells) at an effector: target ratio of 1:1, 5:1, and 10:1 with control IgG or YS110. Anti-tumor activity of effector cells was evaluated by measurement of luciferase activity from the target cells.

## Slide 5
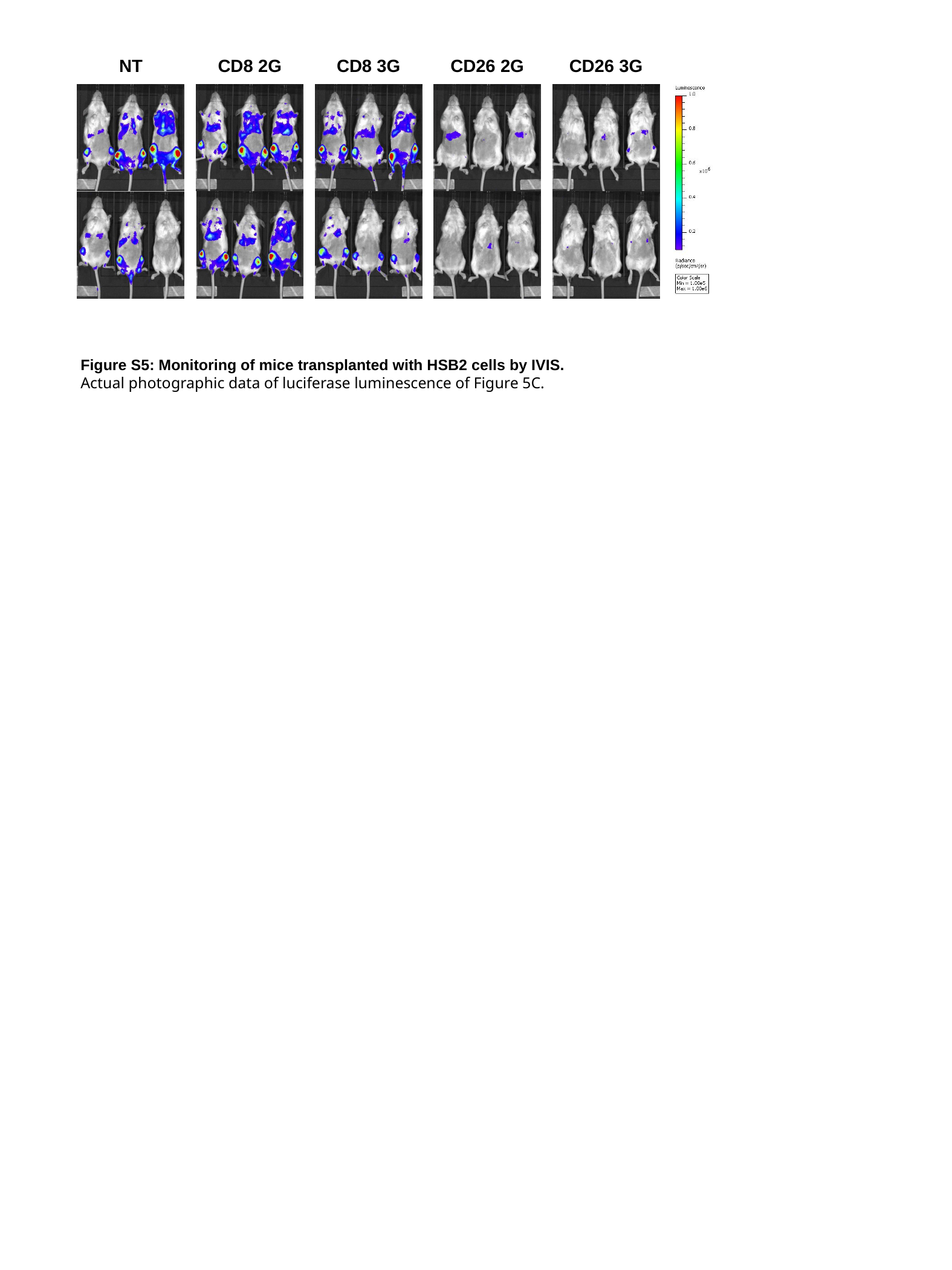

NT
CD8 2G
CD8 3G
CD26 2G
CD26 3G
Figure S5: Monitoring of mice transplanted with HSB2 cells by IVIS.
Actual photographic data of luciferase luminescence of Figure 5C.

## Slide 6
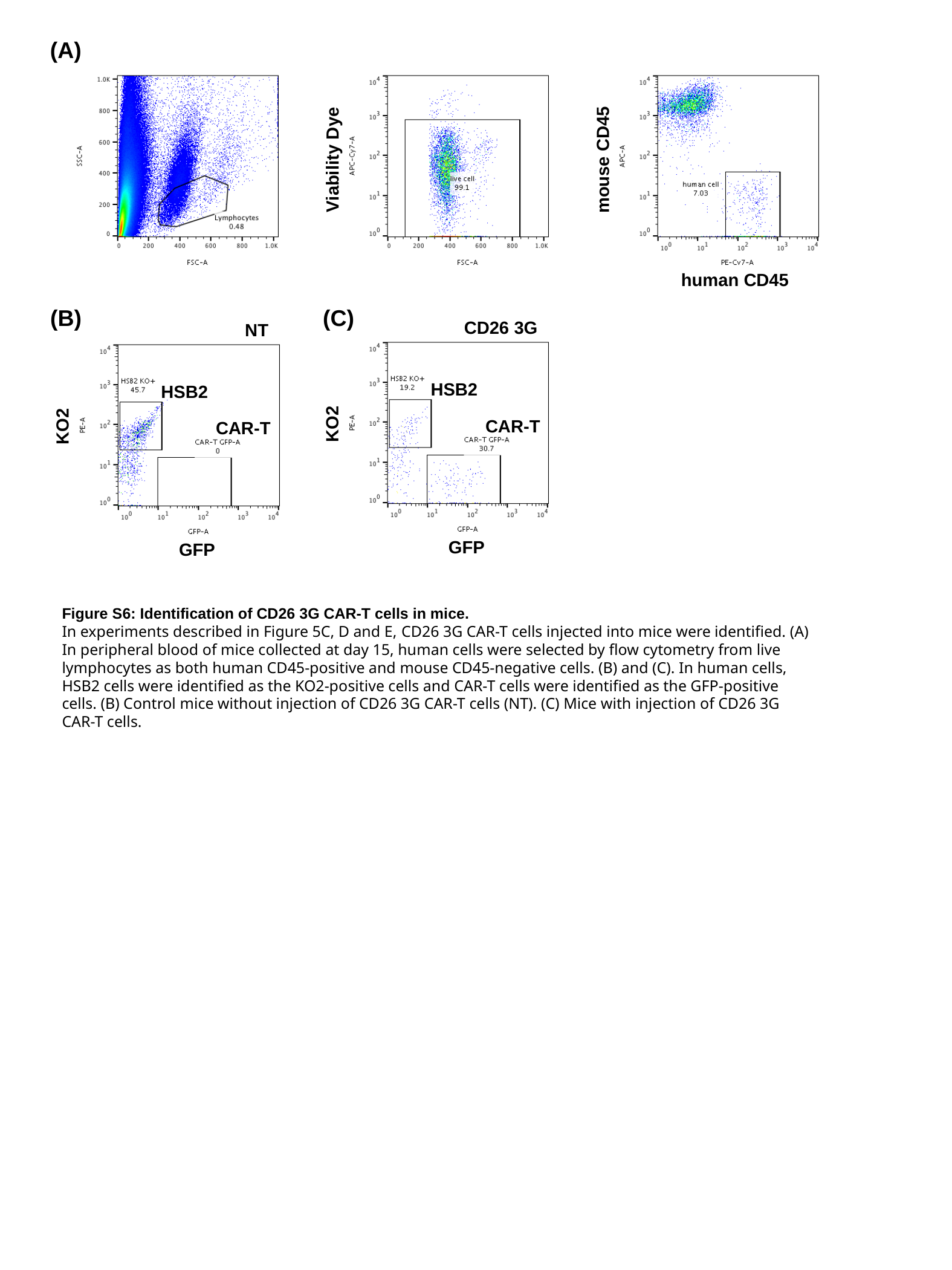

(A)
mouse CD45
Viability Dye
human CD45
(B)
(C)
CD26 3G
NT
HSB2
HSB2
KO2
CAR-T
KO2
CAR-T
GFP
GFP
Figure S6: Identification of CD26 3G CAR-T cells in mice.
In experiments described in Figure 5C, D and E, CD26 3G CAR-T cells injected into mice were identified. (A) In peripheral blood of mice collected at day 15, human cells were selected by flow cytometry from live lymphocytes as both human CD45-positive and mouse CD45-negative cells. (B) and (C). In human cells, HSB2 cells were identified as the KO2-positive cells and CAR-T cells were identified as the GFP-positive cells. (B) Control mice without injection of CD26 3G CAR-T cells (NT). (C) Mice with injection of CD26 3G CAR-T cells.

## Slide 7
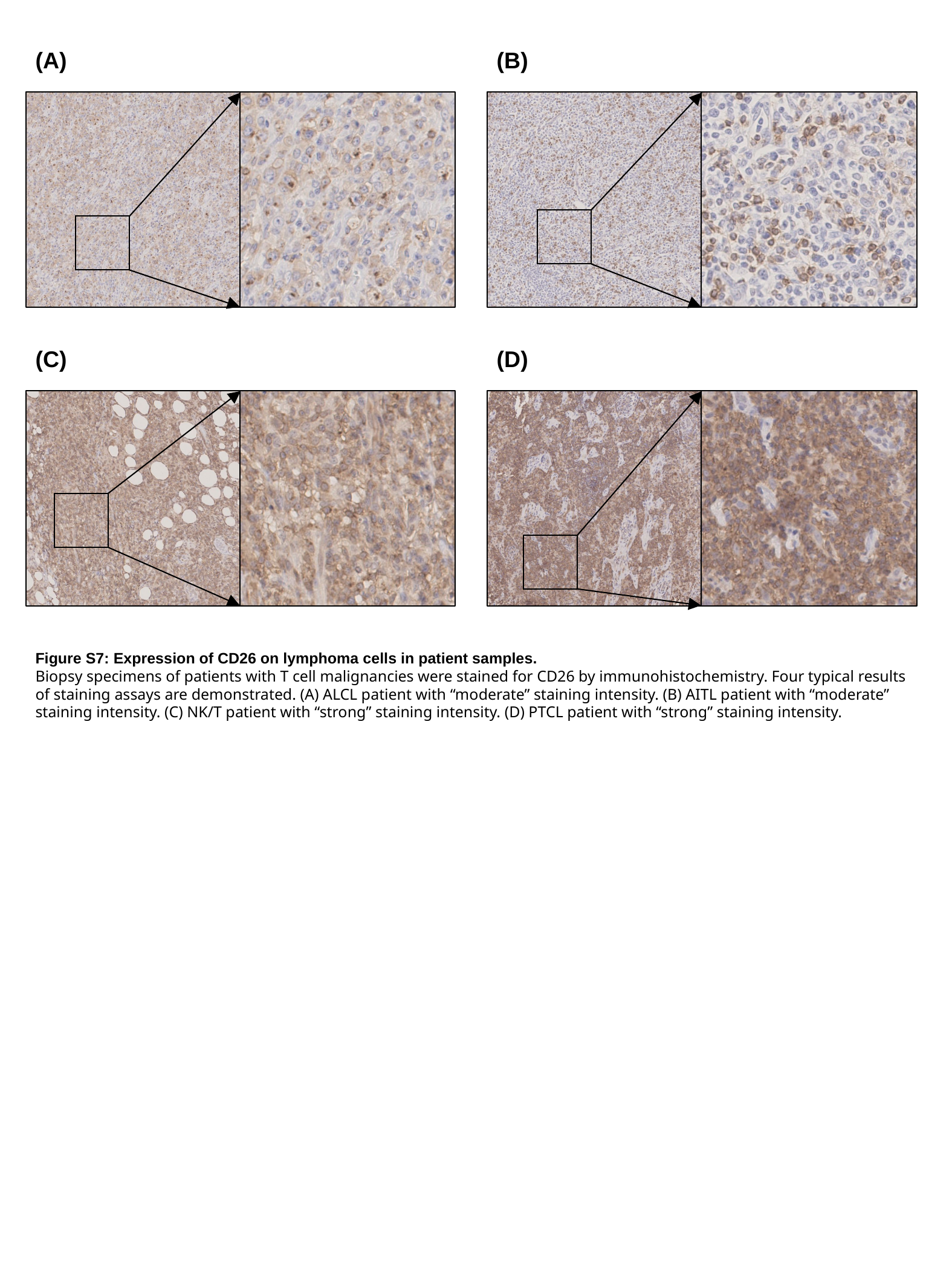

(A)
(B)
(C)
(D)
Figure S7: Expression of CD26 on lymphoma cells in patient samples.
Biopsy specimens of patients with T cell malignancies were stained for CD26 by immunohistochemistry. Four typical results of staining assays are demonstrated. (A) ALCL patient with “moderate” staining intensity. (B) AITL patient with “moderate” staining intensity. (C) NK/T patient with “strong” staining intensity. (D) PTCL patient with “strong” staining intensity.

## Slide 8
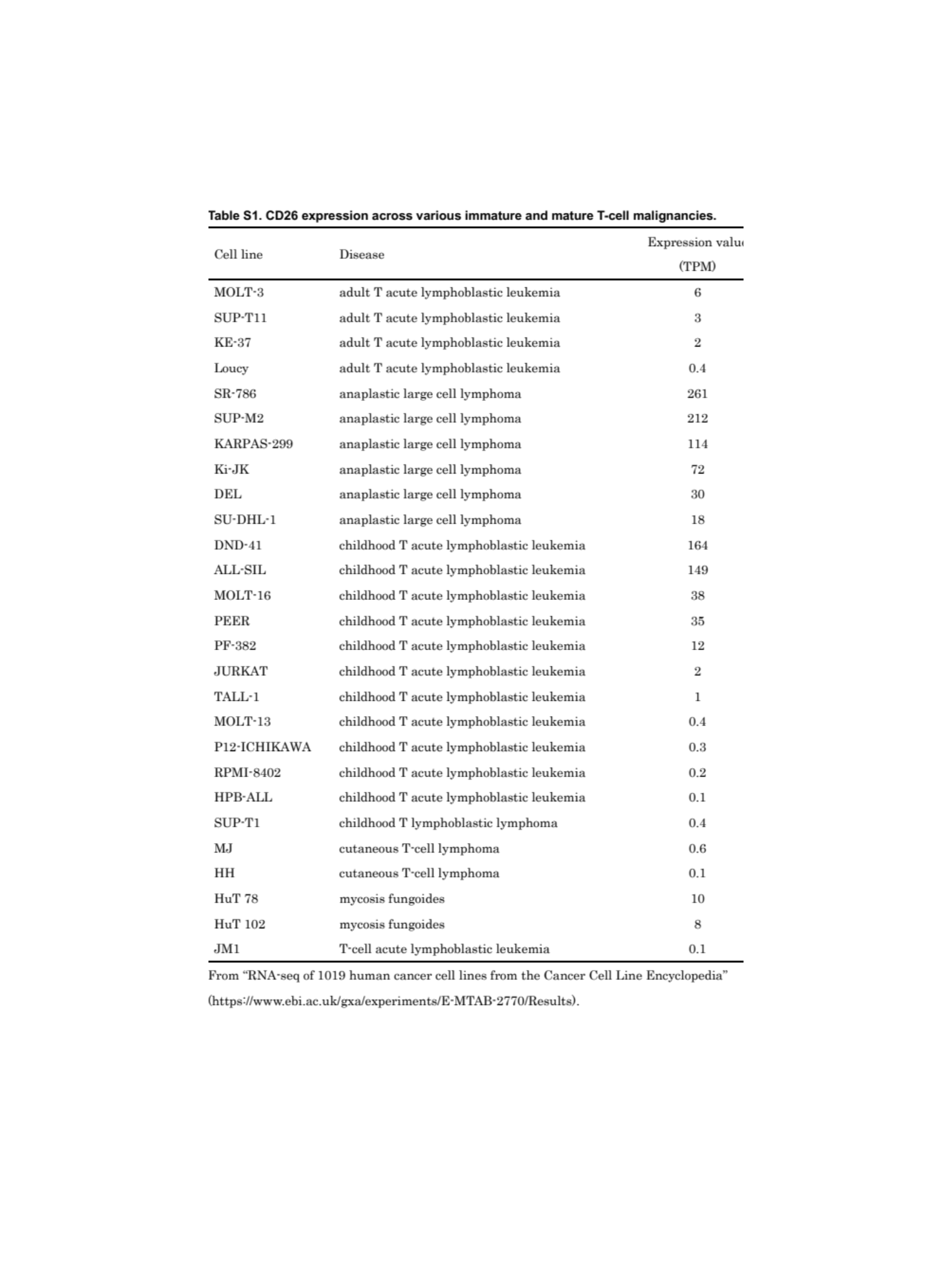

Supplement: Supplementary file 1 [file cells-12-02059-s001.zip › Supplementary information 01.pptx]
